# Supplementary material for: Trends in per Capita Food and Protein Availability at the National Level of the Southeast Asian Countries: An Analysis of the FAO’s Food Balance Sheet Data from 1961 to 2018
Source: Nutrients. 2022 Jan 29;14(3):603. doi: 10.3390/nu14030603 (PMC8838905; doi:10.3390/nu14030603)
Supplement: Supplementary file 1 [file nutrients-14-00603-s001.zip › nutrients-1527656-supplementary.pdf]

Supplementary Tables

Table S1. Summary of the food and protein availability at the national level of the SEA countries.

	Energy				Protein			
	**1961**	**2018**	**Changes [1]**	**AAPC [2]**	**1961**	**2018**	**Changes [1]**	**AAPC [2]**
**Maritime SEA**								
Brunei [3]	1926	2985	54.98	1.2 (0.9-1.5) *	46.15	93.41	102.41	0.9 (0.6-1.0) *
Indonesia	1824	2884	58.11	0.9 (0.6-1.1) *	35.22	67.34	91.20	1.2 (1.0-1.4) *
Malaysia	2418	2845	17.66	0.4 (0.2-0.5) *	46.86	77.45	65.28	1.0 (0.7-1.2) *
Philippines	1788	2662	48.88	0.8 (0.5-1.0) *	41.42	62.54	50.99	0.8 (0.5-1.1) *
Timor-Leste	1741	2287	31.36	0.5 (0.2-0.7) *	62.32	58.02	-6.90	-0.1 (-0.2-0.0)
**Mainland SEA**								
Cambodia	1913	2492	30.27	0.5 (0.1-0.9) *	41.72	65.95	58.08	1.0 (0.3-1.6) *
Laos	1953	2758	41.22	0.5 (0.3-0.7) *	49.28	76.93	56.11	0.7 (0.3-1.1) *
Myanmar	1449	2673	84.47	1.1 (0.9-1.4) *	37.46	90.64	141.96	1.4 (1.2-1.6) *
Thailand	1942	2804	44.39	0.7 (0.4-1.0) *	42.30	63.36	49.79	0.6 (0.3-0.8) *
Vietnam	1907	3025	58.63	0.8 (0.5-1.2) *	45.47	98.58	116.80	1.3 (1.1-1.6) *
**SEA region [4]**	1836	2828	54.03	0.8 (0.6-0.9) *	39.54	73.19	85.10	1.1 (0.9-1.2) *

[1] Values of the percentage changes in food and protein availability at the national level of the SEA countries are given as an absolute figure; directions of the changes from 1961 until 2018 are reported with the + (increased; when availability increased from the intake level of 1961) or - (decreased; when availability decreased from the intake level of 1961) sign; 1961 is considered as a baseline year and 2018 is the latest and last available year. [2] AAPC is the average annual percent change in per capita food and protein availability at the national level of the countries, calculated as a geometric weighted average of the calculated APCs of various segments from 1961 to 2018; in parentheses, the 95% confidence interval is presented. [3] Per capita food availability trend for Brunei Darussalam was analyzed from 1961 to 2013. The energy availability data for Brunei Darussalam was not available in the FAOSTAT new food balance sheet database from 2014 to 2018 while the data was analyzed. [4] SEA region- Southeast Asia region; food availability trends was analyzed from 1961 to 2018 for the SEA region. Per capita food availability data in the SEA region was calculated from the entire ten countries database from 1961 to 2013. From 2014 to 2018, the food availability database in the SEA region did not include Brunei Darussalam as the data was not available in the FAOSTAT food balance sheet database. * Denotes that the AAPC was significantly different from 0 for a specific trend ($P < 0.05$) in the per capita food and protein availability trends of the SEA countries. Values are based on the average annual percent change of the different segment of the trend from 1961 to 2018, considering the introduction of new food balance sheets since 2014 by using the Jump model.

Table S2. Share of plant protein availability at the national level of the SEA countries in different trends period.

	**Trend 1**		**Trend 2**		**Trend 3**		**Trend 4**		**Trend 5**		**Trend 6**	
	Period [1]	% [2]	Period [1]	% [2]	Period [1]	% [2]	Period [1]	% [2]	Period [1]	% [2]	Period [1]	% [2]
**Maritime SEA**												
Brunei [3]	1961-1975	54.14	1975-1984	48.35	1984-2013	47.62						
Indonesia	1961-1963	85.72	1963-1995	83.39	1995-2003	77.29	2003-2018	71.79				
Malaysia	1961-1972	68.11	1972-1978	61.22	1978-1989	51.67	1989-1993	45.08	1993-2018	44.34		
Philippines	1961-1982	59.18	1982-1985	61.27	1985-2018	59.54						
Timor-Leste	1961-1984	52.53	1984-1998	65.57	1998-2018	68.83						
**Mainland SEA**												
Cambodia	1961-1973	86.32	1973-1976	82.92	1976-1991	84.18	1991-1997	80.78	1997-2001	75.48	2001-2018	71.10
Laos	1961-1966	87.81	1966-1986	87.28	1986-1998	85.08	1998-2001	81.65	2001-2018	78.46		
Myanmar	1961-1973	79.65	1973-1984	80.64	1984-1989	78.49	1989-1999	81.85	1999-2011	70.25	2011-2018	56.95
Thailand	1961-1968	71.70	1968-1988	67.47	1988-1996	59.13	1996-2009	59.93	2009-2018	58.91		
Vietnam	1961-1970	79.35	1970-1977	82.30	1977-1986	82.79	1986-1991	79.69	1991-1997	77.08	1997-2018	65.48
**SEA region** [4]	1961-1991	76.24	1991-1995	71.53	1995-1999	70.69	1999-2018	65.35				

[1] Trends analysis identified the specific period of time, which is segment between two adjacent joinpoints where line segment of the food availability (kcal/day/person) trends of the Southeast Asian countries are joined. Food availability trends of the Southeast Asian countries were analyzed on the basis of the Jump model where the effect of the introduction of the new food balance sheet since 2014 was considered. [2] The mean percentage values are given for a specific period of time.

Table S3. Share of animal protein availability at the national level of the SEA countries in different trends period.

	**Trend 1**		**Trend 2**		**Trend 3**		**Trend 4**		**Trend 5**		**Trend 6**	
	Period [1]	% [2]	Period [1]	% [2]	Period [1]	% [2]	Period [1]	% [2]	Period [1]	% [2]	Period [1]	% [2]
**Maritime SEA**												
Brunei [3]	1961-1975	45.86	1975-1984	51.65	1984-2013	52.38						
Indonesia	1961-1963	14.28	1963-1995	16.61	1995-2003	22.71	2003-2018	28.21				
Malaysia	1961-1972	31.89	1972-1978	38.78	1978-1989	48.33	1989-1993	54.92	1993-2018	55.66		
Philippines	1961-1982	40.82	1982-1985	38.73	1985-2018	40.46						
Timor-Leste	1961-1984	47.47	1984-1998	34.43	1998-2018	31.17						
**Mainland SEA**												
Cambodia	1961-1973	13.68	1973-1976	17.08	1976-1991	15.82	1991-1997		1997-2001	24.52	2001-2018	28.90
Laos	1961-1966	12.19	1966-1986	12.72	1986-1998	14.92	1998-2001	18.35	2001-2018	21.54		
Myanmar	1961-1973	20.35	1973-1984	19.36	1984-1989	21.51	1989-1999	18.15	1999-2011	29.75	2011-2018	43.05
Thailand	1961-1968	28.30	1968-1988	32.53	1988-1996	40.87	1996-2009	40.07	2009-2018	41.09		
Vietnam	1961-1970	20.65	1970-1977	17.70	1977-1986	17.21	1986-1991	20.31	1991-1997	22.92	1997-2018	34.52
**SEA region [4]**	1961-1991	23.76	1991-1995	28.47	1995-1999	29.31	1999-2018	34.65				

[1] Trends analysis identified the specific period of time, which is segment between two adjacent joinpoints where line segment of the protein availability (g/day/person) trends of the Southeast Asian countries are joined. Protein availability trends of the Southeast Asian countries were analyzed on the basis of the Jump model where the effect of the introduction of the new food balance sheet since 2014 was considered. [2] The mean percentage values are given for a specific period of time.

Trends in Per Capita Food and Protein Availability at the National Level of the Southeast Asian Countries: An Analysis of the FAO's Food Balance Sheet Data from 1961 to 2018

Supplementary Figures

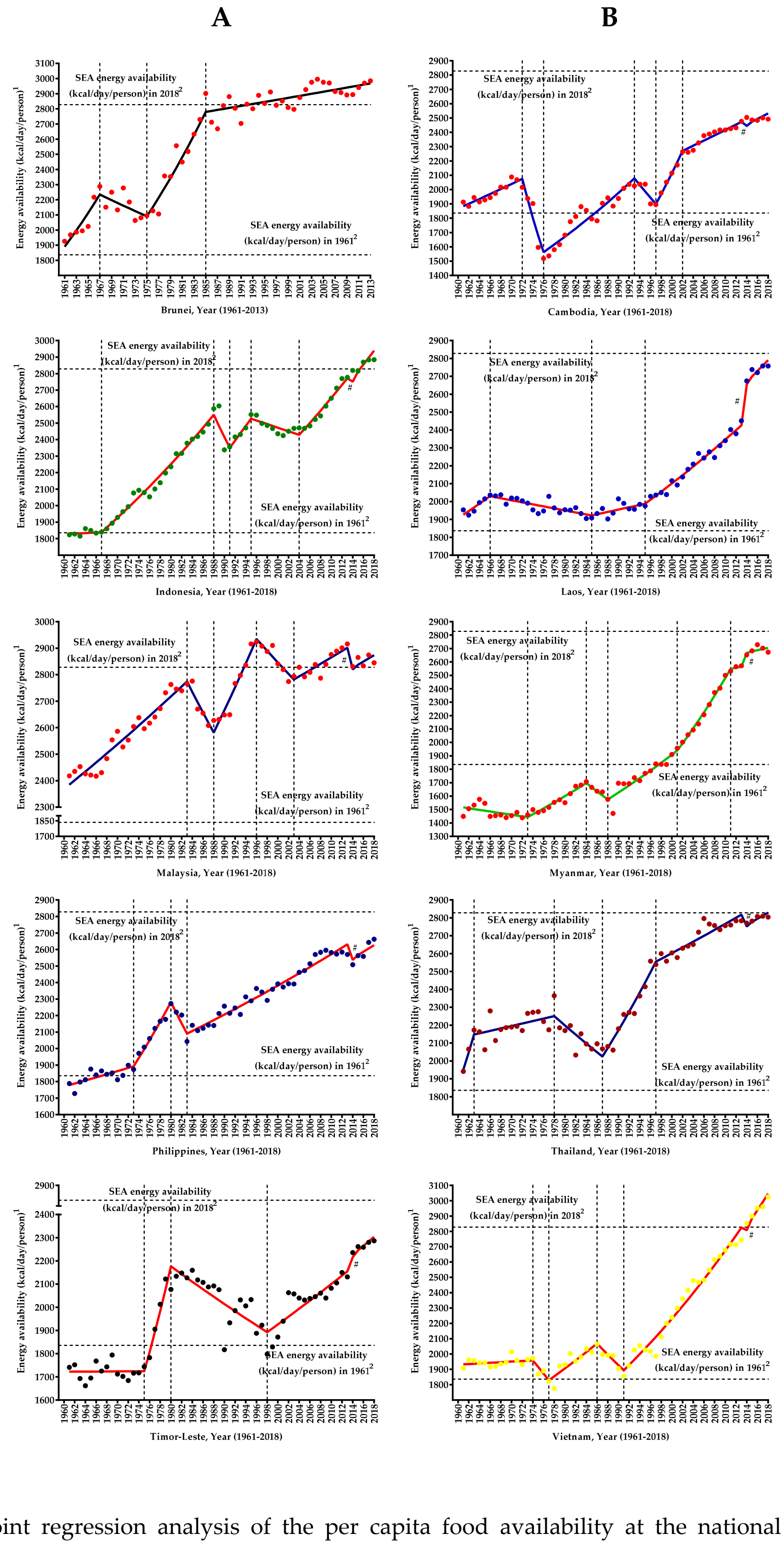

Figure S1. Joinpoint regression analysis of the per capita food availability at the national level of the SEA countries from 1961 to 2018. Panel (**A**) represents the maritime SEA Countries and Panel (**B**) represents the mainland SEA Countries. A vertical dotted line represents the joinpoints. ¹ Food availability (kcal/person/day) refers to the per capita daily calories availability at the national level of the SEA countries. ² A horizontal dotted line represents the energyfood availability in the SEA region during 1961 and 2018. # denotes the location of the jump point due to the introduction of updated FBS since 2014 with the previous FBS (1961 to 2013).

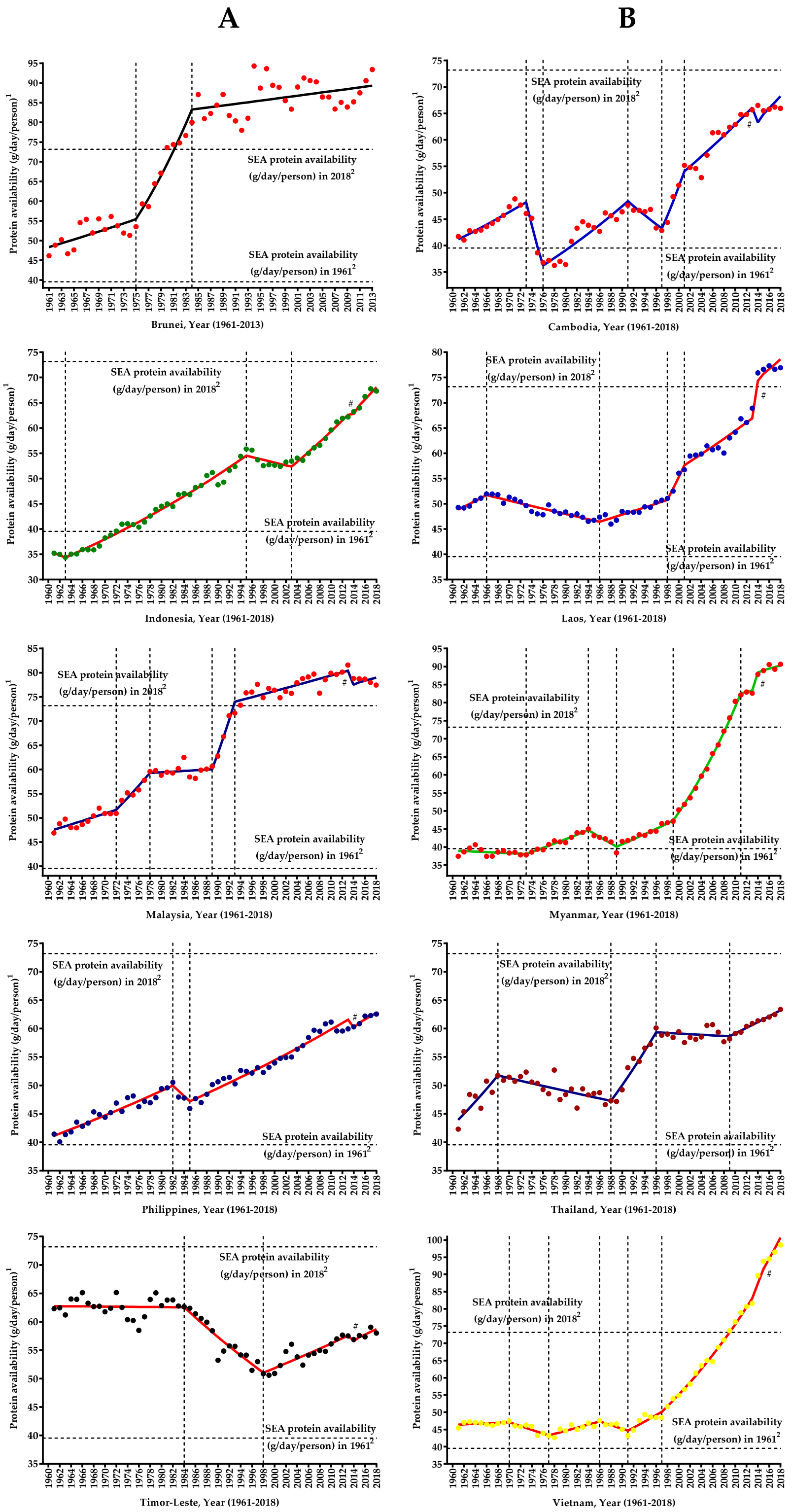

Figure S2. Joinpoint regression analysis of the per capita protein availability at the national level of the SEA countries from 1961 to 2018. Panel (**A**) represents the maritime SEA Countries and Panel (**B**) represents the mainland SEA Countries. A vertical dotted line represents the joinpoints. [1] Protein availability (g/person/day) refers to the per capita daily protein availability at the national level of the SEA countries. [2] A horizontal dotted line represents the protein availability in the SEA region during 1961 and 2018. # denotes the location of the jump point due to the introduction of updated FBS since 2014 with the previous FBS (1961 to 2013).
